# Health-Related Quality of Life (HRQOL) Measures: There are Still Many Unanswered Questions about Human Life

**DOI:** 10.1100/tsw.2008.54

**Published:** 2008-04-14

**Authors:** Mayowa Ojo Owolabi

**Affiliations:** Department of Medicine, University College Hospital, PMB 5116, 200001, Ibadan, Nigeria

**Keywords:** life, quality of life, QOL, health-related quality of life, HRQOL, patient-centered medicine, holistic medicine, brain, mind

## Abstract

Health-related quality of life (HRQOL) measures are used to assess the multifaceted impact of disease, and determine the utility and associated disability. In addition, the impact of medical interventions must be assessed by psychometrically robust HRQOL measures based on a comprehensive and dynamic model. To develop such a model, the concepts of life, its quality, domains, essence, and purpose must be properly and clearly understood. The correct understanding of these entities is specifically important for patient-centered medicine and has universal implications for all fields of human endeavor. Therefore, in order to explore questions about life and quality of life adequately, every necessary field of knowledge should be employed. A multilinguistic and etymological appraisal reveals that life is related to medicine, freedom, being, soul, and spirit, all of which must therefore be considered in its conceptualization.

## INTRODUCTION

### Quality of Life vs. Health-Related Quality of Life (HRQOL)

Colloquially, "quality of life" is a generic term with wide applications and implications in the fields of medicine, economics, political science, and sociology. The focus of this paper is to explore this concept in relation to medicine, i.e., health-related quality of life (HRQOL).

### Importance of HRQOL Measures

HRQOL measures are used to assess the multifaceted impact of diseases on patients' lives and to determine the utility, futility, and disability (challenges) associated with specific disease states. They are also useful in identifying and prioritizing areas of therapeutic needs of individual patients, besides identifying patients with special needs[1,2,3,4].

In the management of diseases, the doctors' duty does not only include the promotion and protection of health and the prolongation and saving of biological lives, but also the improvement of quality of life (i.e., adding meaning to life and adding life to years, and not just years to life). Therefore, the impact of preventive, therapeutic, palliative, and rehabilitative interventions must be assessed by psychometrically robust HRQOL measures based on a comprehensive, rock-steady, and dynamic model. Steady and dynamic like a tree that grows.

## The Need for a Model of Man's Life

For an HRQOL instrument to have adequate content validity, and be parametric of such outcomes as disability paradox "x" and response shift "y", it must be able to assess all domains of life[4]. Likewise, for diagnosis, therapy, and prognosis of diseases to be adequate and complete, it must involve all domains of life. Therefore, to develop a robust and meaningful instrument for measuring HRQOL, the concepts of life, life's quality, domains, essence, and purpose must be properly and clearly understood[5,6,7,8,9,10].

The correct modeling of these concepts is fundamentally important and universally relevant as exemplified by possible applications in the fields of patient-centered medicine, holistic medicine, end-of-life care ("*transition*" *medicine*), and translational medicine (which translates basic and clinical research into HRQOL improvement)[5,6,7,10,11,12,13,14,15,16,17,18,19,20,21]. Other relevant fields are palliative care; medical humanities and philosophy; ethics of abortion, euthanasia, and cloning; medical robotics, artificial intelligence, and human-machine interface; and therapy with neuroimplants[11,13,14]. These concepts are also applicable in cognitive neurology and analytical neuropsychology[5,6,7,11,12,13,14,22].

Neither medical nor psychiatric training provides a good background for the conceptual and terminological approach required in this subject[8,9,12]. In fact, training in biomedicine is likely to produce impatience with the philosophical discourse in this area. Nevertheless, since science is the systematic pursuit (search and research) of the knowledge of the unknown, every necessary field of knowledge should be employed to explore the unanswered questions about life and quality of life[8,9].

## Unanswered Questions Pertaining to Life and Medicine

The neural networks and circuitry for different aspects of human behavior have been identified and examined by novel functional neuroimaging and brain-computer interface techniques, but several questions remain unanswered (see Table 1)[15,16,23]. For example, can we see the mind just as the brain is seen with the physical eye[8,24,25]? What is the basis of extrasensory perceptions, near-death experiences, out-of-body experiences (OBEs), and spontaneous paranormal experiences[9,26,27,28,29, 30,31,32,33,34,35,36,37,38,39]? Is it in the mind or the brain? In OBEs, which are said to occur in one out of every 10 people with many hundred spontaneous OBE stories detailed on the OBE website, which aspect of man observes the other as another body[17,29,30,31,32, 33,34,35,36,37,38,39]?

What is the difference between the mind and the body? What makes man? Is it thoughts or body? How does either relate to life? What is life? If health is the condition for life and disease the condition for death, is health merely the absence of disease[4] or is life merely the absence of death?

To what extent are life, health, disease, and death predicted by the genome[40]? Why do individuals with similar genetic probabilities for a disease have different expressions or no expression of the disease? For instance, monozygotic twins with 100% identical genes have different expressions of diseases such as schizophrenia, multiple sclerosis, and rheumatoid arthritis[41,42,43]. If we could decipher every gene and the functions of each gene, could we understand who would acquire schizophrenia, heart disease, or become a violent person[41,42,43]? *"Gathering knowledge and understanding is a good thing, but we need a bit of humility. We are not just a collection of genes*[40,41,42,43,44]." Why do people react differently to the same disease with different HRQOL profiles even when the disease has the same physical expression (phenotype) and they have the same physical resources to combat it[29,30,45]?

**TABLE 1**
**Unanswered Questions**

| | |
|---|---|
| 1. | What is life? What happens at death and after death? |
| 2. | What is the purpose of life? |
| 3. | What is quality of life? |
| 4. | What is the role of destiny, freewill, and programming in understanding health and disease, life and death? |
| 5. | To what extent are life, health, disease, and death predicted by the genome?[40] |
| 6. | To what extent can neuroimaging decipher the mind?[23] |
| 7. | Mind and brain: are they identical or different entities? |
| 8. | What is the mechanism of spontaneous out-of-body experiences? |
| 9. | What is the neurophilosophical basis of paranormal experiences? |
| 10. | What roles must spirituality play in the principle and practice of medicine? |
| 11. | What is the correct explanatory model for “disability paradox”? |
| 12. | What are the mechanisms of the placebo effect, spontaneous remission, and faith healing? |

Apart from genotypic and phenotypic programming, are there other parallel forms of programming? What roles do the programming of life, beliefs, and indoctrinations; the trajectories and projections of individual life in the “Cartesian matrix of life”, man’s specific and relative experiences; the wounding and the healing of life forces play in the futility and the utility of life? What is the role of cause and effect; and action and reaction (do unto others what you want them to do unto you: compare Newton’s third law of motion) vis-à-vis predestination and freewill in the equation of life? How do these factors contribute to the purpose of life?

What is the purpose of life? How do we tell the worth (quality) of a man’s life? Is it by how much he gains or gives? Is it by how much he builds or buys? Is it by how he feels or what he tells? How can the concepts of predestination and freewill that influence human behavior and reactions to health and disease states be explained? What are the mechanisms and effects of psychotherapy, spontaneous remission, faith healing, and placebo effect on the healing process[17,29,30,35,36,37,38,39]? What is the primal cause of life and death, health and disease?

These important abstract concepts cannot be assessed by literal and literary means (Arabic contributions to arguments in epistemology : *ain-ul-yaqĩn* – certainty by personal inspection) alone, they can only be fully understood, appreciated, and apprehended by including a priori, sublime, and cogitative assays (*ilm-ul-yaqĩn* – certainty by reasoning or inference) in our analysis. The answers to these multidisciplinary questions and concepts would be primary to any analysis that should culminate in a multidimensional quality of life model.

## LIFE

The most pivotal of these concepts is life; hence, it will be explored first. Life, whose understanding is fundamental to medicine, poses a multidisciplinary question. Addressing its conceptualization in all ramifications then provides a standpoint for highlighting its essence, purpose, and quality.

### Etymological Considerations

An etymological and multilinguistic approach reveals that life is body (**lîf)** in old English, ***lîb*** in old high German (also related to *lib*erty and book as in *lib*rary), ***vida*** in Spanish (***veda*** in Indian means to know), and ***zwhn*** in Greek(Table 2)[4]. Is it therefore true that life is a *body (zone)* of *liberty* whose degree of freedom

**TABLE 2**
**AN Etymological Appraisal of "Life"**

| Language | Related Term | English Translation |
|---|---|---|
| Latin | *vivo* | Life |
| French | *vie* | Life |
| German | *das Leben* | Life |
| Old English | *lîf* | Body |
| Old high German | *lîb* | Life; related to *lib*erty and *lib*rary |
| Spanish | *vida* | Life; *veda* in Indian means to know |
| Ancient Indian science of medicine | *ayurveda* | Which means the knowledge of life (*ayu* = life, *veda* = know) |
| Yoruba | *ìyè*<br>*ààyè*<br>*orí inú*<br>*ẹlẹ̀da* | Life<br>Life, chance, free space<br>Equivalent to soul, *chi* in Igbo language<br>Equivalent to spirit of life within |

is determined by the quantum of its embedded *knowledge*? Life is ***vivo*** in Latin, ***vie*** in French, ***das Leben*** in German, ***chay*** in Strong Hebrew, and ***chi*** in Igbo[4,46,47]. Furthermore, in French, the statement of individual life and being "I am" is ***Je suis*** (compare ***Jesus*** "I am… life")[48].

In India, according to the Veda, life is related to the ***ãtmã*** (compare *atom* – "indivisible unit", individual, *automa*tism), which is regarded as a nonphysical, fundamental, indivisible entity characterized by consciousness (wakefulness and awareness). It is the eternal self that resides temporarily in an ephemeral biological form[49].

Notably, the ancient Indian science of medicine is known as **ayurveda,** which means the knowledge of life (**ayu** = life, **veda** = know)[4]. Among the Dinka, ***cieng*** is well-being ("well-Being"), which is intimately associated with ***wei*** ("life", "soul"). Incidentally, life is ***ìyè*** or ***ààyè*** in Yoruba (which also means chance, free space) and the secret word for life in Yoruba is ***ogùngùn*** (àkàrà *ogùngùn* could mean bread of life), which is also the word for medicine and is related to terms such as ***ẹmi*** (spirit) and ***ayé àkamarà*** (earth/world). Other intimately related Yoruba terminologies are ***orí inú*** (equivalent to soul, *chi* in Igbo), ***ẹlẹ̀da*** (equivalent to spirit of life within), ***orí òde/ orí ìta*** (life outside), ***ìwa*** (being), and ***ẹhin ìwa*** (being that persists after the death of the body, afterlife). From the foregoing, life is related to medicine, knowledge, freedom, being, soul, and spirit, all of which must therefore be considered in the search for the secret of life[37,38,50,51,52].

## Reductionistic and Materialistic Considerations

Albert von Szent-Györgyi Nagyrapolt, the 1937 Nobel laureate in physiology or medicine, said *"In my search for the secret of life, I ended up with atoms and electrons, which have no life at all. Somewhere along the line, life has run out through my fingers. So in my old age, I am now retracing my steps…"*[53]. Niels Bohr, the1922 Nobel laureate for physics, also considered consciousness (a characteristic/gnomon of life) thus *"We can admittedly find nothing in physics or chemistry that has even a remote bearing on consciousness. Yet all of us know that there is such a thing as consciousness, simply because we have it ourselves. Hence consciousness must be part of nature, or, more generally, of reality which means that, quite apart from the laws of physics and chemistry, as laid down in quantum theory, we must also consider laws of quite a different kind"*[53,54].

From these statements and the multicultural, multilinguistic, etymological appraisal above, it is obvious that life, in its essence, cannot be reduced to a mere collection of atoms and molecules as it is closely related to the concepts of healing and medicine, consciousness, being and afterlife, freedom and knowledge, and spirit and soul[8]. Physical scientists know that in spite of the theories of the "primordial soup", the neo-Darwinian Jacques Monod concept of life resulting from chance, the Miller's experiment, and the "wonders" of the computer and cloning age[49,53,55], **true** life cannot be artificially synthesized from nonliving entities alone.

Protolife cannot be concocted from a mere localized molecular assemblage because it must autonomously regenerate, reproduce (not merely polymerize), and build new functionality through the evolution of synergistic containment, metabolic and genetic systems for memory (heredity), and novelty (evolution)[11,13,14,56,57]. In the transition from nonliving matter to protolife, inheritable information must take control of the thermodynamic self-assembly and energy transduction processes of glassy systems[11,13,14,56,57].

Thus the proposed "bottom-up" artificial protolife is not **true** life because a unique information system and integrating principle with ever-living purpose and consciousness are still required. Neither is artificial intelligence (AI) equivalent to life as it lacks selfness (individual unique identity and inwardly oriented purpose), self-consciousness, self-determination, self-generation, self-regeneration, self-replication/reproduction, evolution of function and forms to promote perpetual self-survival. However sophisticated, it is still "garbage in, garbage out".

In conclusion, biological life supersedes AI and the "bottom-up" protolife. Thus life, even in its simplest biological form (the smallest viral particle), must be considered beyond the confines of reductionistic materialism[19,54].
